# Large quantum alphabets with a tiny footprint

**DOI:** 10.1038/s41377-024-01550-x

**Published:** 2024-08-23

**Authors:** Fazilah Nothlawala, Andrew Forbes

**Affiliations:** School of Physics, University of the Witwatersrand, Johannesburg, South Africa

**Keywords:** Optics and photonics, Quantum optics

## Abstract

High-dimensional quantum states are known to offer advantages over their two-dimensional qubit counterparts, but their preparation and manipulation has been bulky and cumbersome. Now, quantum state control has been demonstrated on-chip with a ~1 μm^2^ footprint and nm-scale features, producing up to eight-dimensional quantum states and ushering in a new route to large quantum information encoding on a small footprint.

The ability to structure quantum wavefunctions by using the spatial degrees of freedom (DoFs) of light has become highly topical of late^1,2^, driven by the promise of increased information capacity per photon, enhanced security, robustness against cloning and resilience against noise^3^. Unfortunately, the toolkit for preparing and manipulating high-dimensional quantum states is very much in its infancy, often employing bulky set-ups that are derived by analogy with their two-dimensional (qubit) polarisation counterparts. Integrated photonic circuits hold tremendous promise to compactify the quantum hardware^4^, a crucial need for deployment in a global quantum network, but to date only classical states have been demonstrated with such technology, for instance, on-chip creation of scalar^5^ and vectorial^6^ states of light’s angular momentum, and even directly at the source for coherent states^7^, but so far not for quantum entangled states.

Reporting in *eLight*, the group of Liang Feng demonstrate that an entangled polarisation *qubit* state can be “upgraded” in dimensionality to a *qudit* by passing one of the two photons through a customised on-chip integrated photonic circuit^8^, as shown graphically in Fig. 1. The on-chip photon is manipulated across two DoFs, orbital angular momentum (OAM) and spin angular momentum (polarisation) for complete angular momentum control, producing a new state that is increased in dimension, courtesy of the tensor product of the Hilbert spaces of each DoF. Amazingly, the quantum coherence and entanglement quality remain intact, allowing the authors to push the performance to eight dimensional three-qubit cluster states, important for quantum computation. Light–matter interactions at the quantum level seldom leave entanglement preserved, but the present work demonstrates a new pathway to manipulate and control such fragile quantum states.

**Fig. 1:**
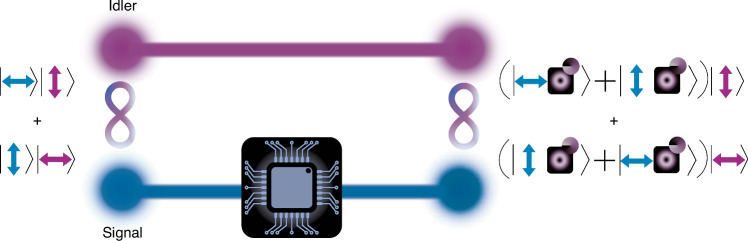
Spin–orbit locked quantum entangled states via a photonic chip. A biphoton polarisation-entangled state is separated such that the signal photon is sent to an integrated photonic chip while the idler photon remains in free-space for remote manipulation of the signal photon at a later stage. The photonic chip converts the polarisation superposition state of the signal photon to a spin–orbit locked superposition state, increasing the dimensionality of this photon to *d* = *4*, while maintaining its original entanglement with the idler photon

The use of spatial DoFs in quantum states can be traced back to the seminal work of Nobel Laureate Anton Zeilinger about 20 years ago, when he demonstrated entanglement in OAM^9^. This opened a path to explore the spatial structure of photons as a large encoding alphabet. Pushing dimensionality increases the information capacity of the structured qudits by Log_2_*d*, where *d* is the dimension of the entangled state (the joint Hilbert space has a dimension of *d*^2^) - an attractive avenue for high-capacity quantum links. Mixing DoFs brings additional benefits, with the most common example being hybrid entangled states of polarisation and OAM^10^, easily created by spin–orbit coupling. The optical elements to execute these advances have typically been very large, e.g., beam-splitters for Bell measurements and liquid crystal devices based on geometric phase for state control; consequently, quantum hardware for structured qudits are bulky and cumbersome. Is it possible to manipulate high-dimensional quantum states on-chip? Here progress has been fruitful in DoFs such as frequency and time, borrowing technology from telecommunications^11^, but such states have no spatial structure to speak of. Doing the same with structured photons has remained elusive due to the seemingly complex task of matching modes of free-space to those on-chip, and controlling patterns of light in highly confined and geometrically restrictive material structures.

To address this challenge, Liang Feng and colleagues begin by producing a biphoton polarisation-entangled state in free-space. The entangled photons are then spatially separated such that the idler photon remains in free-space for later manipulation of the signal photon, while the signal photon is directed to the photonic circuit through two waveguides, following the conversion of its polarisation DoF to a path DoF. The signal photon’s superposition state is thus converted from a polarisation superposition to a superposition between the two on-chip waveguide modes, $|{1}_{s} >$ (paired with waveguide mode $|{2}_{s} >$) and $|{3}_{s} >$ (paired with waveguide mode $|{4}_{s} >$). Four on-chip phase shifters are used to control the relative phase and amplitude between the modes. The four waveguide modes are then sent to two on-chip microring resonators with angular gratings (inscribed on the inner sidewall of the rings) which structure the signal photon into free-space Bessel modes with spin–orbit locking. This spin–orbit locking of the signal photons means that for a given polarisation state of the photon, the OAM state is fixed, and vice versa. The photonic chip is thereby used to increase the dimensionality of the signal photon to *d* = 4, while preserving its original entanglement with the idler photon. Consequently, the spin–orbit state of the signal photon becomes entangled with the polarisation state of the idler photon, resulting in a multidimensional entangled state.

Building further on this, the authors harness the entanglement between the two photons to completely reconfigure the signal photon’s state in the spin–orbit space, forming a high-dimensional SU(4) higher-order Poincaré sphere (HOPS) for the signal photon. By post-selecting the polarisation states of the idler photon across its entire Poincaré sphere, they sample a range of superposition and basis states for the signal photon. This diverse set of states enables the construction of a complete high-dimensional HOPS for the signal photon, encompassing all possible superpositions in the combined polarisation and spatial mode space. Moreover, the researchers showcased the viability using this multidimensional entangled system with spin–orbit qudits for quantum communication by simulating the transmission of an encrypted image under the high-dimensional BB84 protocol. An image is encrypted using the quantum keys as outlined in the BB84 protocol, sent to the receiver and decoded by the receiver’s secret keys. In their paper, the authors show that the image is successfully decrypted, illustrating the robust capability of secure quantum communication using entangled spin–orbit states. Notably, using the 4D flying qudits allowed transmission and encoding of twice as much information, compared to traditional qubits, all while maintaining the same single photon generation rate.

The possibility of integrating quantum technology into the established platforms for fast and efficient on-chip control of classical light is highly appealing. It would allow fully integrated quantum systems with fibre optics, for instance, and could pave the way to sophisticated quantum gates for high-dimensional states. Liang and colleagues present a very promising example of just such a situation, bringing the tools of structured light, quantum optics and integrated circuits to a fruitful embrace. In the future it is possible to integrate the source of the entangled photons on-chip too, and to push the dimensionality higher. This paints a clear and promising path to flying structured photons with high information capacity yet on a tiny footprint for integrated deployment, e.g., for satellite quantum communications, where size, speed, robustness and efficiency are paramount.

Historically, high-dimensional structured quantum states have lingered in laboratory demonstrations, far behind the satellite and terrestrial deployment of polarisation qubits. Now, Liang Feng and colleagues have introduced a novel toolkit for unprecedented quantum control that leaps beyond the present status quo, opening a new pathway to reconfigurable quantum circuits that are large in functionality but small in size.

## References

[1] Nape, I. et al. Quantum structured light in high dimensions. *APL Photonics* **8**, 051101 (2023). 10.1063/5.0138224

[2] Erhard, M., Krenn, M. & Zeilinger, A. Advances in high-dimensional quantum entanglement. *Nat. Rev. Phys.* **2**, 365–381 (2020). 10.1038/s42254-020-0193-5

[3] Forbes, A. et al. Quantum cryptography with structured photons. *Appl. Phys. Lett.* **124**, 110501 (2024). 10.1063/5.0185281

[4] Elshaari, A. W. et al. Hybrid integrated quantum photonic circuits. *Nat. Photonics* **14**, 285–298 (2020). 10.1038/s41566-020-0609-x PMC8607459 34815738

[5] Bütow, J. et al. Generating free-space structured light with programmable integrated photonics. *Nat. Photonics* **18**, 243–249 (2024). 10.1038/s41566-023-01354-2

[6] Zhong, H. Z. et al. Gigahertz-rate-switchable wavefront shaping through integration of metasurfaces with photonic integrated circuit. *Adv. Photonics* **6**, 016005 (2024). 10.1117/1.AP.6.1.016005

[7] Forbes, A., Mkhumbuza, L. & Feng, L. Orbital angular momentum lasers. *Nat. Rev. Phys.* **6**, 352–364 (2024). 10.1038/s42254-024-00715-2

[8] Zhao, H. Q. et al. Integrated preparation and manipulation of high-dimensional flying structured photons. *eLight* **4**, 10 (2024). 10.1186/s43593-024-00066-6

[9] Mair, A. et al. Entanglement of the orbital angular momentum states of photons. *Nature* **412**, 313–316 (2001). 11460157 10.1038/35085529

[10] Forbes, A. & Nape, I. Quantum mechanics with patterns of light: progress in high dimensional and multidimensional entanglement with structured light. *AVS Quantum Sci.* **1**, 011701 (2019). 10.1116/1.5112027

[11] Zhang, Z. S. et al. Entanglement-based quantum information technology: a tutorial. *Adv. Opt. Photonics* **16**, 60–162 (2024). 10.1364/AOP.497143

